# Visitation, communication, and participation: an ethical and respectful strategy for engaging Australian South Sea Islander communities when sharing their lived experiences

**DOI:** 10.3389/fsoc.2024.1372727

**Published:** 2024-09-12

**Authors:** Francis Bobongie-Harris, Zia Youse, Gordon Quakawoot

**Affiliations:** ^1^Faculty of Creative Industries, Education and Social Justice, School of Early Childhood and Inclusive Education, Queensland University of Technology, Brisbane, QLD, Australia; ^2^Faculty of Arts and Sciences, University of Sydney, Sydney, NSW, Australia

**Keywords:** Australian South Sea Islanders, decolonising methodologies, Tok Stori, engagement strategy, archaeology

## Abstract

This study examines the experiences of three Australian South Sea Islanders from the same community who are part of a larger research team scheduled to engage in an archaeological research project in Mackay, Queensland, in 2021. The aim of the project was to highlight Australian South Sea Islander knowledge, voices, and lived experiences. However, owing to COVID-19 and persistent lockdowns in Queensland and New South Wales, the project faced significant delays. The timeline was adjusted, with plans to reintroduce the project to the community after an 18-month hiatus, aiming to rekindle interest and encourage participation. This study focuses on designing a community engagement strategy that builds on established protocols for researchers working with Australian South Sea Islander communities. The strategy includes three key steps: visitation, communication, and participation. By integrating the Tok Stori methodology—an Indigenous research approach from the Solomon Islands and Vanuatu—the strategy supports the decolonisation of the collaborative research process, enabling the sharing of Australian South Sea Islander lived experiences in a culturally safe manner. The authors provide contextualised reflections, offering insights from an Australian South Sea Islander perspective.

## Introduction

The sugar cane farms in the Pioneer Valley at Mackay are a physical reminder to Australian South Sea Islanders of an industry their ancestors established. The first South Sea Islanders were brought to Mackay in 1867, from Vanuatu, known as New Hebrides at the time. Mackay had the largest Pacific Islander population in Australia, with 40 sugar mills in operation at one point and was also the largest sugar-producing area in Australia (Moore, 2001). Australian South Sea Islanders are descendants of South Sea Islanders and comprise a unique and culturally diverse community.

This study highlights the importance of cultural processes and protocols when connecting and engaging with Australian South Sea Islander communities, both as community members with insider knowledge and as outsiders and a part of a larger research team. It introduces a three-step engagement strategy: visitation, communication, and participation. This engagement strategy was trialled and employed by Australian South Sea Islander researchers, utilising the practice of Tok Stori an Indigenous research method and dialogical practice synonymous with the Solomon Islanders and Vanuatu, to encourage the sharing of shared and lived experiences of the Australian South Sea Islander community and more specifically the Homebush Mill Estate. This study primarily focuses on the cultural requirements and processes involved to ensure that researcher engagement with Australian South Sea Islander communities is both respectful and ethical.

Before we begin, and as Australian South Sea Islander researchers, it is important to position ourselves within the community and the research project, ensuring that those that we work with know our ontological connections, relationships, and how to relate to us. We are diverse in our representation of ancestry from the Solomon Islands and Vanuatu. Our Australian South Sea Islander identity and our cultural perspectives are a part of who we are. We invite you to listen as we “stori” (tell stories) and “tok” (talk) about our identity and who we are.

*FBH*: I identify as an Australian South Sea Islander through my great-grandparents, grandfather, and father. I am a third- and fourth-generation Australian, and my ancestors are from the islands of Malaita in the Solomon Islands. I was born in Mackay where you will find the largest Australian South Sea Islander community in Australia. I have had the opportunity to return to the Solomon Islands where I have made strong family and cultural connections. Up until COVID-19, I would visit the Solomon Islands once a year to maintain my family relationships and connections. We remain close even though we are far apart.

*ZY*: I identify as a third-generation Australian South Sea Islander with a heritage from the islands of Ambrym, Buka, Gaua, Lifou, Malaita, and Malekula. I was raised in Mackay, amongst many other South Sea Islander families. Unlike my grandparents, I have not returned to the islands and have yet to make that cultural reconnection.

*GQ*: I identify as a second-generation Australian South Sea Islander. My grandfather on my father’s side is from Buka, Western Solomon Islands. My grandfather on my mother’s side is from Malaita, Solomon Islands. I have not been to the Solomon Islands, but I have been to Vanuatu on several occasions. Before my first visit, my mother had reconnected with her father’s family when she visited Vanuatu. She would keep in contact with them through writing and passed on photos of her family here to family that she had met in Vanuatu. When I followed my mother, in making my first trip to Vanuatu, I knew that was my family line.

*FBH*: The shared histories between the Solomon Islands and Australia are of interest to me as they are part of my DNA, and they are part of my identity. I grew up with similar cultural traditions to that of my family in the Solomon Islands. These cultural traditions were passed down to me by my grandfather and my father. These were more evident as a child growing up when we would have visitors from the Solomon Islands and later when visiting the Solomon Islands. Weddings and funerals were important events, as was going to church every week. Getting together with everyone always led to storytelling and sharing of information; genealogy and cultural knowledge that has been passed down through each generation.

*ZY*: My Islander culture was strengthened through events that brought South Sea Islander families together. One of those was attending church on Saturdays. From a child, this tradition became the backbone of my Islander culture. In Mackay, we would often attend two churches that had a large number of South Sea Islander people. Cultural connections were also maintained through family gatherings such as reunions, parties, weddings and funerals. The preparation that went into organising those events involved a lot of family meetings where we would eat together and talk about family genealogy. The stories passed down would be shared time and time again, and now these have become my stories. These events, along with travelling to visit South Sea Islander families in other towns of Queensland, as a means of staying connected, are an important part of my culture as an Australian South Sea Islander.

*GQ*: Family was and still is my culture. As a child, my family would go to get-togethers down on the river where our families lived. We, the kids, would play while our aunties washed their clothes in the fresh water and rubbed them against the rock with soap. When there was not sufficient food, families would go up the creek for taro with old hessian bags and cane knives. In addition, my father and uncles would go fishing. This food would then be shared between the families. At these times, I would listen to the stories told of the “old boys”. The “old boys” referred to are those men who came out from the islands (the Solomon Islands and Vanuatu) to work the cane. They would talk of which islands the “old boys” came from and what they did here. These stories would be repeated at many get-togethers and that is how they have been passed on.

As Australian South Sea Islander researchers, collectively, our connections to the South Sea Islander community of Homebush, are through family and church. Drawing on these connections was an important part of the engagement strategy and will be discussed throughout the article.

## Homebush Mill Estate

The research project began its first archaeological dig at the site that once housed Homebush Mill located south of Mackay in April 2020. In 1882 the Colonial Sugar Refining Company (CSR) built this extensive and complete mill (Munro, 1895). The estate mill had a crushing capacity of 9,000 tons and was the largest in the district, using machinery and techniques that were the equal of any in the world (Munro, 1895; Moore, 1974).

In the 1890s, with the limited continuation of Pacific Island labour and easier land laws affecting small agricultural farms, many white immigrant labourers looked for a future in small-crop cane farming. CSR Homebush Mill had 31 small farmers supplying cane from 755 acres (Moore, 1974). The first farmer to lease land from CSR Homebush in 1894 was JH Taylor, who lived in a two-roomed weatherboard and iron house, had a four-stall stable, a chaff house, a dray shed, “Kanaka” quarters, and a 20-foot bricked well (Munro, 1895; Moore, 1974).

## Homebush and South Sea Islanders

One of the earliest recorded connections of South Sea Islanders to CSR at Homebush Estate involves 50 men brought over on the labour-recruiting vessel “Chance” (Noel Butlin Archives Centre, Australian National University, 1882). The estate employed South Sea Islander labourers alongside Cingalese, Chinese, Javanese, and Japanese workers.

**Figure 1:**
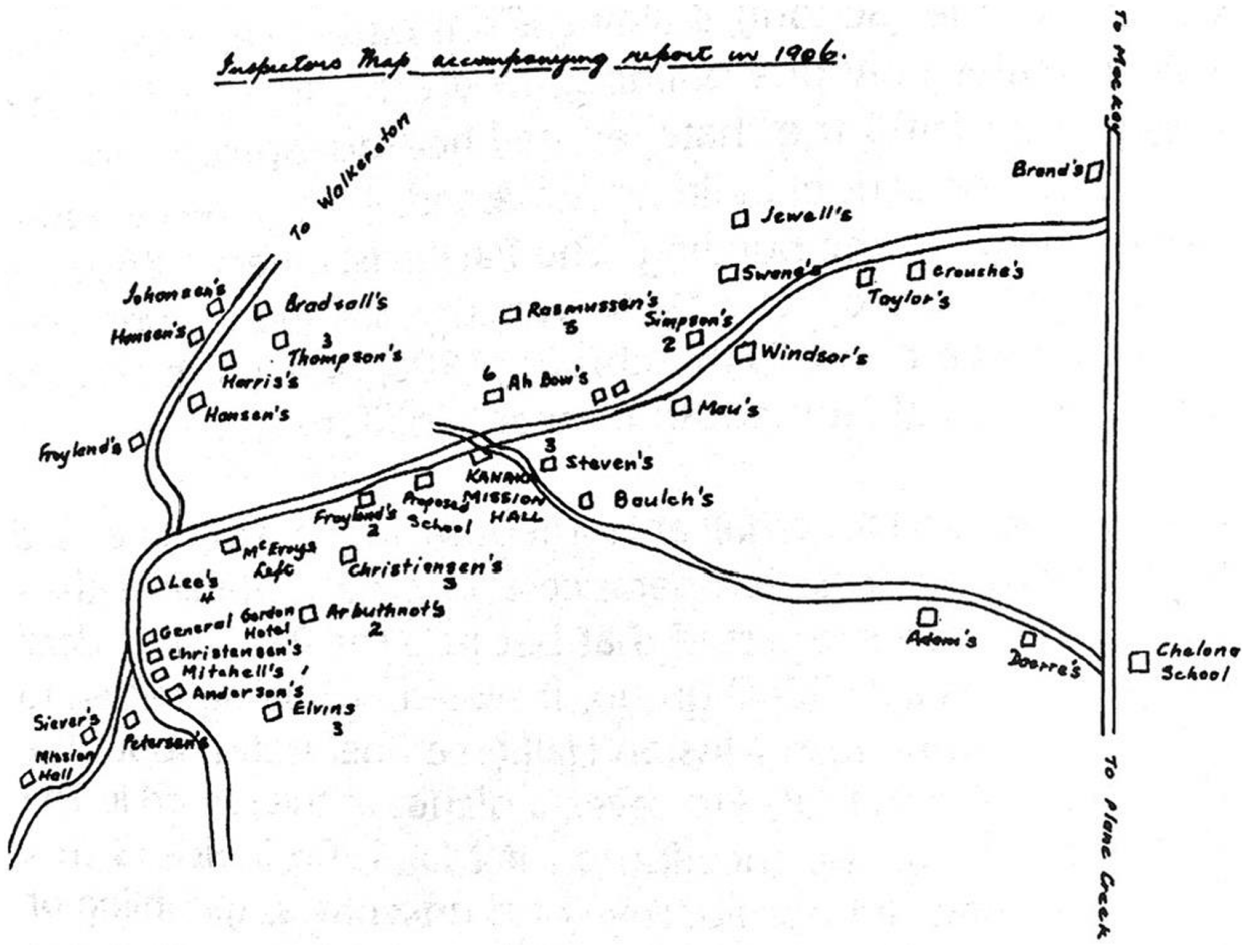
Map drawn by Mr. A Mutch, who was the District Inspector of Schools at the time (Hatfields Printers, 1975, p.25).

Beyond their labour, South Sea Islanders attended mission classes in a schoolhouse they built on land allocated by the CSR Company at Sandiford, near Homebush (Daily Mercury, 1907). The Kanaka Mission School, adjacent to the Kanaka Mission Hall, served religious and educational purposes until the mass deportation (Daily Mercury, 1907). The remaining South Sea Islanders then attended the present-day Homebush Mission Hall. Figure 1 identifies the Kanaka Mission Hall at Sandiford and the present-day Homebush Mission Hall, noting the adjoining schoolroom known as the Kanaka School (Andrew and Kennedy, 1995; Daily Mercury, 1907).

The rural township of Homebush and the old Homebush Mission Hall are significant to the history of South Sea Islander peoples. Many families endured labour in the sugar industry, with some returning to their island homes or other places of employment, while others settled in Homebush. The present-day Mission Hall is the last remaining physical link for South Sea Islanders of the area. Historically, it was a spiritual meeting place for the Andrew, Arrow, Baggow, and Thomas families. Andrew and Kennedy (1995) document these families’ fond experiences, highlighting their spiritual and communal connection to the Mission Hall.

Cedric Andrew recalls his early memories: “I went to Sunday School and the Church Service till the Mill closed up in 1922… We came up, the three of us, Grandad, Grandmother, and myself in a dog cart… Sunday school was at 9 o’clock and the Church Service was after… I was seven years old when the 1918 cyclone destroyed the [first] Mission Hall” (Andrew and Kennedy, 1995, p. 6).

Colleen Baggow also shares her memories: “The Mission Hall at Homebush was the place I first went to Sunday School with my two sisters and also nieces and nephews… All of my six children were christened there by the serving minister of that time. I myself was christened there… When the children were growing up, they went to Sunday School there too” (Andrew and Kennedy, 1995, p. 8).

The Homebush Mission Hall was heritage-listed by the State of Queensland in 1997 and is occasionally used for commemorative events, such as the 160 years of Australian South Sea Islanders in Australia, and as a place of worship (Queensland Government, 2022). It remains an official meeting place for Australian South Sea Islanders in the area, restored and maintained by volunteers from the Australian South Sea Islander community (MADASSIA, 2000), some of whom still reside in Homebush.

## Decolonising research

As Australian South Sea Islander researchers, our insider perspectives challenge Western epistemologies regarding our community and disrupt settler narratives about the country, the community, and its history. The lived experiences of South Sea Islanders associated with the old Homebush Mill were pivotal to the archaeological research project. Our connections to the Australian South Sea Islander families from Homebush endowed us with unique insights specific to our community. This insider knowledge enabled us to decolonise our practice, and the narratives presented in the research project, both from within and later as external observers.

### Research framework

The research project’s structure presents a modified version of Bobongie-Harris et al. (2021) “Country, Community and Indigenous Research Framework”. The framework adapts to the Australian South Sea Islander community residing on the ancestral lands of the Yuwi people and guides our engagement strategy. Yuwi Country, also known as Mackay, is a coastal city in Queensland, Australia, established in 1862 with sugar as its economic cornerstone. This historical context of sugar cultivation forms the basis for the deep connections of Australian South Sea Islanders to Yuwi Country, through relationships and connections with its people, place, and community. Our research utilises a community-based research approach with the inclusion of Tok Stori a Melanesian Indigenous research method. The research is grounded in our comprehensive understanding and the interconnectedness of these elements.

## Acknowledgement of country

All research conducted in Australia, whether Indigenous or non-Indigenous, is situated on Aboriginal and/or Torres Strait Islander Land, referred to as “Country.” This research project was conducted in Yuwi Country, the Traditional Lands of the Yuwi people. According to the Australian Bureau of Statistics (2021), two out of three Australian South Sea Islanders also identify as Aboriginal and/or both Torres Strait Islanders through marriage, thereby establishing their own connections to the country. In recognition of these connections, we acknowledge the Traditional Custodians of Yuwi Country and pay our respects to their elders past, present, and emerging.

## Place and people

South Sea Islanders were Indigenous to their own lands before their arrival in Australia, maintaining customs and traditions that connected them to their ancestral lands and communities. Upon their arrival in Australia, they brought a rich diversity of Melanesian Culture from the Solomon Islands and Vanuatu, which remain integral to Australian South Sea Islander life and continue to shape engagement processes with various Australian South Sea Islander groups (Moore, 2001). Australian South Sea Islander culture is a blend of the old and the new and includes those customs and traditions from their islands of origin, developed by association with the wider Australian community (MADASSIA, 2000).

## Research methods

### Community-based qualitative research approach

The project utilised a collaborative community-based qualitative research approach, emphasising active community member involvement (Duke, 2020). Globally, archaeologists have embraced this method, particularly in Indigenous, racialised, and vulnerable communities. Lewin (1946) pioneered action research with Jewish communities in New York, advocating for involving community members as co-researchers to address real-world problems and empower participants. Similarly, Tax (1958) demonstrated the collaborative and transformative potential of research with the Meskwaki Tribe in Iowa (Foley, 1999). Thompson’s work in Guam highlighted the importance of trust and mutual respect between researchers and community members, crucial for meaningful and impactful research (Duke, 2020).

Atalay (2012), an Ojibwe archaeologist, significantly influenced Indigenous archaeology in North America by advocating for community-based research. This approach emphasises ethical and sustainable practices by involving communities in the research process, recognising Indigenous expertise, and moving beyond theoretical frameworks to practical applications. In post-native title Australia, Buhrich et al. (2019) underscored the value of a collaborative research approach. Engaging communities as active partners in archaeology fosters a sense of ownership and respect, ensuring that research addresses community needs and perspectives. Boyd (2020) stressed the necessity for researchers to be flexible and responsive to community needs and priorities. This flexibility is essential for conducting ethical and effective research, especially with vulnerable populations. A collaborative, community-based approach not only enriches research but also fosters stronger, more respectful relationships between archaeologists and the communities they study, producing accurate data and promoting empowerment for marginalised communities.

Overall, this approach significantly enhances the relevance and ethical integrity of archaeological projects. By actively involving community members, particularly those from vulnerable groups, the research process becomes more inclusive and respectful. This leads to more meaningful and sustainable outcomes, ensuring that the research addresses real-world problems and respects the perspectives and needs of the communities involved.

This research project is one of a few archaeological endeavours in an Australian South Sea Islander community using a collaborative, community-based approach led by non-Australian South Sea Islanders. Preparing the community and encouraging participation required significant groundwork. We aim to present an Australian South Sea Islander perspective on engagement based on our insider knowledge and everyday practices within our community, which remain consistent whether in research or not.

### Tok Stori as an indigenous research methodology

Tok Stori is a Melanesian Indigenous research methodology characterised by its dialogical nature, integral to everyday conversational communication in the Solomon Islands and Vanuatu. This method is inherently flexible, adapting to the dynamic needs of Melanesian life. As Sanga and Reynolds (2018, p. 17) describe, “In Tok Stori, relationality, information, and time come together to form a distinct way of being.” It involves speakers and listeners becoming part of each other’s narratives, fostering deep connections (Sanga et al., 2020a,b). Recognised as a culturally acceptable way of sharing information (Vella and Maebuta, 2018), Tok Stori is traditionally used for intergenerational discussions (Brigg et al., 2015). As Australian South Sea Islanders, we regularly engage in these dialogical intergenerational discussions, maintaining our cultural heritage and ties to the Solomon Islands and Vanuatu. This methodology ontologically connects Australian South Sea Islanders to their Melanesian origins.

### Integrating Tok Stori and community-based research

Integrating Tok Stori into a community-based research approach leverages the strengths of both methodologies, resulting in a more inclusive, culturally sensitive, and effective research process. This integration yields richer data, stronger community engagement, and more impactful outcomes. Within the Australian South Sea Islander context, this alignment is particularly relevant in four key areas:

1. Emphasis on relationships: Both methodologies prioritise relationships, creating safe spaces for sharing stories and building trust and collaboration (Sanga and Reynolds, 2023; Sanga et al., 2020a,b).
2. Participatory nature: Both are inherently participatory, encouraging active involvement and equal contribution from participants (Bolinga, 2023; Friendship et al., 2012).
3. Contextual flexibility: Tok Stori adapts to specific cultural and social contexts, aligning with community-based research’s goal to address community-identified problems meaningfully (Bolinga, 2023).
4. Empowerment of participants: Both methodologies empower participants by facilitating knowledge sharing and building community capacity to address their issues (Sanga et al., 2020a,b; Friendship et al., 2012).

Engaging community members as active participants ensures culturally relevant, ethically conducted, and beneficial research, especially within the Australian South Sea Islander community of Homebush. This approach focuses on understanding historical and lived experiences, ensuring cultural sensitivity and direct community benefits.

## Our roles as researchers

A researcher’s cultural background and relationships significantly influence their position within the research, allowing them to identify as insiders and/or outsiders (Bobongie-Harris, 2021; Kerstetter, 2012). Insiders, such as those within the Australian South Sea Islander community, possess deep insights into community dynamics due to their upbringing and cultural practices. Conversely, as part of a broader research team, researchers must balance Western research norms that emphasise emotional distance with the necessity of maintaining trust within their community (Mercer, 2007; Kerstetter, 2012).

Research roles are fluid (Hanza et al., 2016), and there is no need for a binary insider-outsider identification (Bucerius, 2013). Operating as outsiders with insider knowledge allows researchers to immerse themselves in the community, leveraging their cultural understanding and established relationships. This dual perspective enhances the research process, particularly in data collection from Australian South Sea Islander participants, demonstrating the value of their knowledge.

*FBH:* I have been awarded research grants that allow me to work in the Australian South Sea Islander space, looking specifically through an educator’s lens. I am conscious that I am privileged to work in this space, and I do not take the sharing of Australian South Sea Islander knowledge for granted. As an outsider: and a researcher in this space, I feel there is an expectation by colleagues to deliver outcomes. As an insider in this space, my community comes first, and my decisions in the inclusion of them in any form of research are determined by their availability and their willingness to engage and then lead. I became a part of this research project by accident. I came across the information well and truly after the funding was awarded and the consultation process had happened. There are very few academics who identify as Australian South Sea Islander researchers and who furthermore engage solely in Australian South Sea Islander research. My role on the project has evolved from that of recording stories to the inclusion of research—that is the history, stories, and objects—in the Australian curriculum. Regardless of my role, I aim to develop my relationships with my community further when working alongside them as part of my practice. Having insider knowledge is important to fulfil my research role as an outsider, as I can understand what the community is happy to share with non-community members and what they are still unsure about. The processes involved in using content from the project in the curriculum require continual guidance and contribution from the broader Australian South Sea Islander community.

*ZY*: I was made aware of this research project by an Australian South Sea Islander friend, FBH who is a Chief Investigator on the project. The project was three years old when FBH asked me if I was interested in becoming a research assistant (RA) on an archaeological research project. I knew I would be stepping into unknown territory, researching in an academic space. However, I was confident to work within my own community of Mackay. As an insider, I am wary of the respect that must be given to the Australian South Sea Islander community with regard to storytelling for research purposes. Even Australian South Sea Islanders within the same community can have differing views about this. As an outsider and part of the research team, I believe it is essential that the Australian South Sea Islander community benefit from the research project by building the capacity of the community and ensuring that mutually agreed outcomes are met.

*GQ:* The project team were travelling to Mackay and needed a logistics coordinator. I do a lot of work within my community and have already driven the project team around Mackay on one of their first visits to the area. FBH and ZY organised an interview via Zoom with the project leader. Following that interview I was hired. Before the arrival of the team, I organised the bus hire and collection. Owing to COVID-19 restrictions, their visit was delayed 1 week. However, at this time, I took it upon myself to connect with my community and visit Australian South Sea Islander significant places. I organised a community bus tour and stories were shared amongst the community. Upon the arrival of the team, I collected them from the airport and drove them to their accommodation. There was a daily schedule; each day, I would drive the team of students out to the Homebush excavation site, collect them at the end of the day, and return them to their accommodation or drive them out for dinner. I also did extra trips collecting equipment for the excavation site, driving students to the library and a trip to the chemist. My role centred mostly around community engagement.

## Community-based research in Australian South Sea Islander communities

Conducting community-based research in Australian South Sea Islander communities using a collaborative approach, requires adherence to specific protocols and guidelines established by the Mackay and District Australian South Sea Islander Association (MADASSIA) in 2000. These protocols ensure respectful and culturally sensitive research practices. MADASSIA (2000, p. 6) states, “If other organisations or individuals are seeking to support or carry out projects that affect the lives of Australian South Sea Islanders, then they are urged to consider the protocols carefully.”

### Initial consultation and community involvement

From the outset, researchers must provide comprehensive information about the project, enabling the community to fully understand the research objectives and processes. Involving the community in planning ensures cultural appropriateness and maximises benefits for the community. Additionally, researchers must provide written feedback and copies of reports generated from the research to participants. MADASSIA (2000, p. 23) advises, “Workshops should be held to support a greater understanding of plans.”

### Community expectations

The MADASSIA (2000) protocols document clearly outlines community expectations:

1. Reciprocity: Researchers should engage in reciprocal relationships with the community, offering employment opportunities, facilitating workshops, and ensuring the research benefits the community (pp. 9, 16, 23, and 24).
2. Health and safety: Researchers should consider the health and safety of community members, providing food that meets dietary requirements and covering associated costs during meetings (pp. 21 and 26).
3. IT proficiency: Effective communication should use various forms and be accessible to all, recognising that not everyone prefers or has access to technological devices (pp. 9 and 10).
4. Informed consent: Obtaining informed consent is a detailed process beginning with community engagement. Initial contact involves formal letters to local organisations, followed by personal outreach. A community engagement gathering allows the community to meet the research team, ask questions, and receive detailed project information. This process provides time for community members to consider participation, discuss with family, and seek further clarification in follow-up meetings (pp. 8, 9, 10, and 26).
5. Inclusion of Australian South Sea Islanders as part of the project: It is imperative to include Australian South Sea Islander community members from the project’s inception, ensuring their active involvement in the management and delivery of the work. Organisations undertaking such projects must allocate a sufficient budget for the costs and wages associated with employing Australian South Sea Islanders. This approach not only promotes community engagement and ownership but also ensures that the research process is respectful and inclusive of the community’s expertise and perspectives. By integrating local knowledge and providing fair compensation, the project can foster a collaborative and empowering environment for all stakeholders (pp. 16, 17, 23, and 26).

Researchers must be flexible, allowing changes where possible, and clear about non-negotiable aspects. By adhering to these guidelines, researchers can ensure that their projects are conducted ethically, respectfully, and in a manner that genuinely benefits the Australian South Sea Islander community of Mackay.

## Research design: community engagement strategy

The research project was conducted over three weeks and comprised three main components: an archaeological dig at the old Homebush Mill site, a community workshop at Mackay Art Space, and the recording of Australian South Sea Islander stories. As Australian South Sea Islanders within the research team, our challenge was to engage our community as research participants.

Australian South Sea Islanders are considered a vulnerable group. As insiders, we felt a responsibility for our families and community, particularly amid the COVID-19 pandemic. We ensured the research team’s compliance with Queensland Health guidelines, including mask wearing and COVID-19 testing, to prioritise community safety.

To maximise community awareness, flyers created by ZY and distributed by GQ included information about a community dinner and visits to the archaeological dig site. These flyers were also shared through the Australian South Sea Islander peak body’s social media platforms and email.

Understanding the community’s needs, we offered them the option to share their stories without pressure to have them recorded. This approach was communicated on the project’s first night, reinforcing that the research team would be available to record stories over the next three weeks, should community members choose to participate (Personal Reflection: ZY, June 2021).

The research design for the project incorporated a “Community Engagement Strategy,” structured into three components aligned with a modified version of the Bobongie-Harris et al. (2021) framework “Country, Community, and Indigenous Research Framework” and includes the protocols and guidelines as set out by MADASSIA:

1. Visitation: Informing community members about the research project through home visits.
2. Communication: Discussing the research project through community sessions and dinners.
3. Participation: Involving community members through bus tours and an organised archaeological field day.

These elements are explained further in the following sections.

### Visitation

The first week was all about the community. As insiders, we knew that visitations were a suitable option to meet in a way that suited community interests. This also aligned with the protocols document: “Support the community to know more about what you are planning and how to plan (MADASSIA, 2000, p. 23)”. Being an elder in the community, it was important that GQ took on this role to visit with the relevant families connected to Homebush as “Elders are respected and are ‘looked after’ by other family members (MADASSIA, 2000, p. 6)”. As insiders, ZY and FBH knew that they should allow GQ to engage in the visitations as part of the Engagement Strategy and resume their roles as support people.

*GQ*: To engage with the community, I prefer to visit people rather than email them or rely on Facebook, especially because many of the older community members are not savvy with emails or do not bother themselves with such technology. My project engagement work began with me delivering flyers to the community and talking to them about the planned archaeology work at Homebush and the Objects Workshop. By personally visiting, people were then able to ask me questions and even willingly shared their stories about the site. Quarterly newsletters were produced by the project team after their visit to Mackay, and I continued to visit the community and deliver the newsletters, ensuring that people were always aware of the project’s plans once the research team left Mackay.

In addition to the visits, two events were planned in a specific way to encourage communication, transparency, and participation by the community: a community dinner and bus tours. Because of the unpredictability of COVID-19, Brisbane went into lockdown, and this delayed the arrival of the team by one week. We decided to keep up the momentum of re-introducing the project back into the community by continuing with both events. This extra time with the community meant that we could draw on the progress that GQ had made with his community visits and get everyone together to talk some more about the project.

### Communication: community dinner and information sessions

As COVID-19 hit Sydney and then Brisbane, the project leader emailed and suggested that GQ and ZY continue to run the planned community dinner with the guidance of FBH and IM (partner investigator and Australian South Sea Islander). The original plan was that a cultural awareness programme be held for the research team and new archaeology students in alignment with MADASSIA protocols. They would be welcomed into the country by Yuwi Traditional Owner Uncle GT and refreshed on the Australian South Sea Islander protocols and procedures by community member DF. This would be accompanied by a dinner.

Get-togethers and sharing a meal are an important part of Australian South Sea Islander culture, so GQ organised for the local Solomon Islander group (who are connected to the Australian South Sea Islander community through marriage) to cater for the evening with traditional island dishes. ZY organised the speakers for the night. To do our best in connecting the community with the project team, ZY asked the project leader, two principal investigators and a research assistant from the Queensland Museum to record videos of themselves that could be presented to the community on the night. Because they were all to work closely with the community and had an interest in the community, they were asked to record a first video that introduced themselves and their role in the research project; the second video was to explain why they think stories are important to this research project.

*ZY*: While I did not doubt that GQ and I could run an information session and community dinner, I did doubt that we could audio record stories as suggested by the project leader. At the outset, I knew it would not be culturally appropriate to record stories at the community dinner, even though the project team had already visited the Mackay Australian South Sea Islander community several times over the past four years. In the collection of stories, it would be more culturally appropriate to let the community know that GQ and I were now on the project team, and they could share their stories over the next three weeks with us and the rest of the team if they would like to. Standing before my community, I felt indebted to them and wanted to assure them that their stories would be collected and used to produce an output that was meaningful for them. As community members shared their memories with me, they expressed their pride that I am on the project to help strengthen our cultural identity for future generations.

### Participation: bus tours

The initial plan for the bus was to provide transportation for the students, academics, and professional staff visiting Mackay for the research project. It would transport them between the work site and accommodation. However, after the bus was booked and COVID-19 changed plans, it became an essential part of the community engagement strategy.

*GQ*: The idea to run community tours on the bus came to me when the research team had to delay their trip for one week, due to COVID-19. I had already collected the hired bus and would have it for a week without the research team. I knew I had to use it for the project, so I decided to run a community tour.

Another community field day was organised to transport community members to the archaeological site at Homebush. It was designed as an information session to inform and update the community about what was happening on-site. In the end, the archaeological dig was abandoned early because asbestos was found at the site and the site was closed. However, because the community workshops were well received by the community, the community continued to participate in the activities, further sharing their stories.

*GQ*: Knowing that the original plans for the archaeology field day had been changed due to asbestos, I wanted to make sure the community would have an opportunity to visit the site and meet the team, to hear more about the project. I organised another community bus tour, inviting the men to come out. Others also jumped on board, and at the end of the tour, many said it was interesting and that they were glad that they went.

### Lived experiences of Australian South Sea Islanders connected to Homebush Mill Estate

The literature review at the beginning of this article provides a historical snapshot of the CSR Homebush Mill Estate and the surrounding areas as recorded by historians. The data were collected from the participants through Tok Stori and provided another account of the CSR Homebush Mill Estate and its surrounding areas through an Australian South Sea Islander lens. These data were collected as part of the bus tours, by ZY during part two of the community engagement strategy.

*GQ*: The first trip came about because of the community night where I parked the bus outside the venue. I had offered to transport people for the night, and at the end of the night, when everyone was sharing stories amongst themselves, a couple of families decided to organise a bus trip to the site. From this, we set a day and then as a family does, they told others, who then told others, and, on the day, we ended up with quite a few more family members and friends. Even now, the family continues to mention the project team, saying, “they said they were coming back.”

*ZY*: On the first community tour, GQ drove the bus around and picked up the community members. I sat at the back of the bus with everyone and gave them the participant information statement and the participant consent form. Once we arrived at the site, we got off the bus and I explained the various parts of the consent form. Most consented to the audio recording, and I then showed them the voice recorder, keeping it in their view to ensure they could remember that I was recording. I also took my children on this first tour as those on the bus were our relatives. In this way, my children could hear the stories being told by elders and see the places of significance to other South Sea Islander families. There were several interactions that took place across the generations. This included the sharing of stories, my eldest son helped the elderly on and off the bus and, the elders consoled my young daughter while I spoke with the community off the bus. This positive engagement has left the community asking, “when am I going to see you again?” which is really encouraging for future community participation.

## Analysis of engagement strategy

Using visitation and communication to encourage participation are not new ways of engaging within the Australian South Sea Islander context. The challenges of COVID-19 within this research context presented an opportunity to formalise and present a collaborative process that demonstrates how to engage respectfully and ethically with the Australian South Sea Islander community. With the implementation of a community engagement strategy that focussed on visitation, communication, and participation, and by establishing clear roles as insiders/outsiders, researchers FBH, ZY, and GQ maintained momentum for the larger research team once they arrived in Mackay, Queensland after being delayed.

The community engagement strategy employed was successful as it kept the Australian South Sea Islander community interested in the research project. The community information sessions and dinners were well attended, and the bus tours were successful with many groups from the community riding initially with ZY and GQ and then with the larger research group once they arrived. Some of the community felt comfortable sharing their experiences about what it was like being an Australian South Sea Islander and growing up in Mackay, with community members connecting stories to the archaeological site (Homebush Mill) and objects shared.

GQ played an integral role in his visitations. His seniority provided access to a generation of elders that he had already established relationships within the community. He was able to connect with the community by providing necessary information about the research work and encouraging participation in community dinners, archaeological field days, and object workshops. This provided opportunities for the larger research team to be able to connect with the community. The Australian South Sea Islander protocols set out by the Mackay and District Australian South Sea Islander Association (MADASSIA, 2000), also guided the researchers and the community to work together in a culturally appropriate way to meet the needs of the community and while collecting rich data, told through an Australian South Sea Islander lens.

## Conclusion

The positive reception and active participation of community members highlight the effectiveness of our engagement strategy. By valuing and incorporating the lived experiences and voices of Australian South Sea Islanders, we not only enriched our research but also empowered the community, ensuring that their knowledge and cultural heritage were honoured and preserved.

As we move forward, the foundations laid by this engagement strategy will continue to guide our interactions and collaborations with the community. The success of this approach underscores the importance of culturally sensitive and community-based methodologies in research, paving the way for future projects that respect and amplify Australian South Sea Islander voices and perspectives. Through ongoing collaboration and the continued application of these principles, we aim to contribute to the decolonisation of research practices creating more inclusive and equitable knowledge production.

The successful implementation of the community engagement strategy in our research with the Australian South Sea Islander community has proven to be a significant achievement. By prioritising ethical considerations, cultural protocols, and the use of the Tok Stori methodology, we were able to foster a respectful and trusting relationship with community members. This trust and mutual respect were evident in the overwhelmingly positive response from the community, who expressed eagerness to continue collaborating with the research team.

“When am I going to see you again?” P.B. 2021.

## Data Availability

The original contributions presented in the study are included in the article/supplementary material, further inquiries can be directed to the corresponding author.
